# Tramadol and the risk of fracture in an elderly female population: a cost utility assessment with comparison to transdermal buprenorphine

**DOI:** 10.1007/s10198-015-0673-1

**Published:** 2015-04-11

**Authors:** Alexander Hirst, Chris Knight, Matt Hirst, Will Dunlop, Ron Akehurst

**Affiliations:** 1 BresMed Health Solutions Limited, Northchurch Business Centre, 84 Queen Street, Sheffield, S1 2DW UK; 2 Mundipharma International Limited, Unit 194, Cambridge Science Park, Milton Road, Cambridge, CB4 0AB UK; 3 School of Health and Related Research (ScHARR), University of Sheffield, Regent Court, 30 Regent Street, Sheffield, S4 1DA UK

**Keywords:** Tramadol, Fractures, Pain, Elderly, Buprenorphine, Cost-effectiveness

## Abstract

**Introduction:**

Opioid treatment for chronic pain is a known risk factor for falls and/or fractures in elderly patients. The latter cause a significant cost to the National Health Service and the Personal Social Services in the UK. Tramadol has a higher risk of fractures than some other opioid analgesics used to treat moderate-to-severe pain and, in the model described here, we investigate the cost effectiveness of transdermal buprenorphine treatment compared with tramadol in a high-risk population.

**Methods:**

A model was developed to assess the cost effectiveness of tramadol compared with transdermal buprenorphine over a 1-year time horizon and a patient population of high-risk patients (female patients age 75 or older). To estimate the total cost and quality-adjusted life years (QALYs) of treatment, published odds ratios are used in combination with the published incidence rates of four types of fracture: hip, wrist, humerus and other.

**Results:**

The model shows tramadol to be associated with 1,058 more fractures per 100,000 patients per year compared with transdermal buprenorphine, resulting in transdermal buprenorphine being cost-effective with an incremental cost-effectiveness ratio of less than £7,000 compared with tramadol. Sensitivity analysis found this result to be robust.

**Limitations:**

In the UK data, there is uncertainty regarding the transdermal buprenorphine odds ratios for fractures. Odds ratios published in Danish and Swedish studies show similar point estimates but are associated with less uncertainty.

**Conclusion:**

Transdermal buprenorphine is cost-effective compared to tramadol at a willingness-to-pay threshold of £20,000 per QALY.

## Introduction

Falls are a major burden to the UK National Health Service (NHS), with an annual cost estimated to be more than £2 billion [1]. The elderly are at particularly high risk and the NHS and the Personal Social Services (PSS) estimate that high-risk groups, such as the elderly, have a high incidence of falls and account for 66 % of the total costs for the UK population [2]. The health burden is expected to increase in line with the average population age. In addition to costs incurred by the NHS, there are broader societal costs that are experienced by individuals and other government agencies. It has been estimated that the NHS accounted for only 59.2 % of the total costs of falls, with a large portion of the remainder being shouldered by patients themselves and their carers [2]. The majority of the health service burden of falls relates to the 10 % of elderly fallers who sustain a serious injury; of those injuries, approximately half will be fractures [3].

To reduce this burden, the National Institute of Health and Care Excellence (NICE) in the UK recommends that elderly patients should receive a multi-factorial falls risk assessment including a medication review with consideration of modification/withdrawal to support risk reduction [4]. The use of psychotropic medications including opioid pain medication is a long established risk factor for falls and fractures in the elderly. Meta-analyses of epidemiologic studies report a moderate increase in the risk of fracture (pooled relative risk 1.32–1.42) for patients on long-term opioid analgesics [5]. Published studies show that higher opioid doses increase the risk of fracture, with the hazard ratio ranging from 1.2 to 5.1, and there are significant risk differences between medications with different active opioid ingredients [6–9].

Opioids are an important pharmaceutical treatment for moderate-to-severe pain. Withdrawal of opioid use without having effective replacement therapies available could lead to worse patient outcomes and create costs for the health system through inadequately controlled pain. An alternative approach to the withdrawal of treatment could be to modify the medication used, thus avoiding opioids that are associated with a high risk of falls and fractures. Two independent studies, in Denmark and the UK, show that patients using tramadol have a higher relative risk of fractures compared with the general population [9, 10]. A third independent published study, in Sweden, shows that the risk of falls increases on initiation of opioid therapy and that tramadol is associated with a higher risk compared with patients who had not received treatment for 3 months [11]. All of these studies are based on extensive government databases and, as such, are a robust source of real-world evidence and comparative effectiveness.

A cost–utility model has been constructed to evaluate the cost and patient utility implications of modifying a patient’s opioid treatment regime from tramadol to transdermal buprenorphine. Our paper focuses on an elderly population at high risk of falls and fractures using the odds ratios from the UK study of fracture risk [10] to explore the impact on absolute fracture incidence of using tramadol compared with transdermal buprenorphine. Other studies have also shown transdermal buprenorphine to be associated with a lower risk of fracture and falls [9, 11]. Transdermal buprenorphine is indicated for moderate pain, similar to the indication for tramadol. Tramadol is the most widely prescribed monotherapy for moderate pain in the UK, transdermal buprenorphine is evaluated as the comparator because it is a commonly used therapeutic alternative to tramadol that is differentiated in terms of mode of action and method of administration. Both treatments are in a similar position in the treatment pathway, and therefore, transdermal buprenorphine is a rational comparator.

We have attempted to be as explicit as possible regarding the model structure and inputs so that readers are in a position to make an informed judgement about its validity and, therefore, the robustness of the results. The goal was to make the model fully replicable. All model inputs are available in the public domain. Many of the inputs for the model were based on a publicly available NICE submission for a therapy that was approved for the prevention of osteoporotic fractures [4]. The full model equations are also provided in this paper.

## Patients and methods

### Overview

Estimates of the incidence of fractures in a population treated with tramadol, a population treated with transdermal buprenorphine and a general population are derived by applying the fracture odds ratios from Li et al. [10, 12] to epidemiological data on fractures in the UK. The population considered by the model is a female population aged 75 or older, as this population was identified as being of high risk for fractures. The health system costs and patient utility outcomes for the extra fractures amongst the opioid-treated populations are used to populate the cost–utility model.

**Fig. 1:**
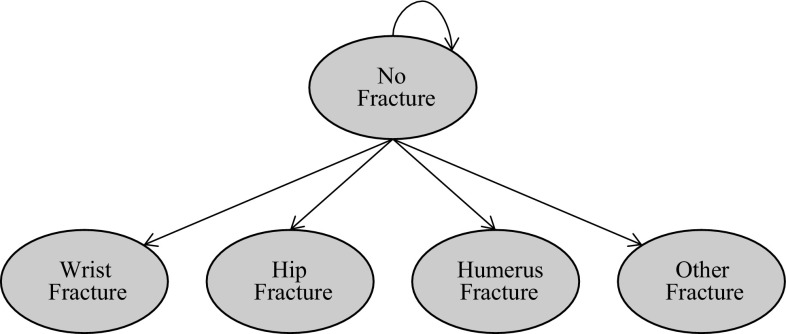
Model diagram—a graphical representation of the micro simulation model

The micro simulation model was developed using Microsoft Excel^®^ 2010. Effectiveness is measured in terms of quality-adjusted life years (QALYs), and costs in British Pounds Sterling (£). The cost utility model looks at costs from the perspective of the UK NHS and PSS over a 1-year time horizon. The final output of the model is an incremental cost-effectiveness ratio (ICER) showing the cost for each QALY gained by using transdermal buprenorphine instead of tramadol. The model diagram is shown in Fig. 1; this was replicated for each treatment in the model and was based on patients starting on pain medication.

### Model population

A source was required to identify the incidence of fractures in a high-risk demographic. The underlying risks of fractures for a general population were collected from a paper by Singer et al. [12]. The risks are used within the model as the probability of moving between health states. Singer et al. was identified, via a systematic review, as the most appropriate source for estimating the underlying fracture risk in a recent NICE submission ([4], Section 6.3.1 in TA204), and we have adopted it as our source for that reason.

The base case examines a high-risk population of female patients aged 75 and older. Todd et al. [3] states that the incidence of falls increases with age and that gender plays a factor in the incidence of fractures in older populations. Singer et al. [12] reports fracture risks by 5-year age groups. To generate a risk of fracture for the general population of women aged 75 and older, these fracture risks are weighted by the general population in each 5-year age category that is also reported by Singer et al. [12].

The same weighted average method is also used for a population of female patients aged 85 and older, which is considered in a sensitivity analysis. This population is at a higher risk of fracture than the default population. Table 1 shows the incidence of fractures in the general population and the odds ratios for fractures in the two opioid-treated patient groups considered within the model.

**Table 1:** Model inputs for fracture incidence in the general population, odds ratios of fracture for opioid patients, treatment costs and utilities

Fracture	Incidence for women in general population 75+ [12] (alpha, beta)	Incidence for women 85+ [12] (alpha, beta)	Odds ratio for transdermal buprenorphine [10] (SE)	Odds ratio for tramadol [10] (SE)	Cost of fracture [4, 18] (SE)	Proportion of patient moving to a nursing home post fracture [4]	Cost of nursing home [4]	Utility multipliers [4] (SE)	Baseline utility [20] (SE)	Health state utility
Equation parameter	*F*_i_	–	OR_i_1	OR_i_2	*C*_i_	PNH_i_	CNH	*U*_i_	BU	
Hip	1.5294 % (517, 33287)	3.0947 % (258.5, 8094)	1.20 (0.48)	1.30 (0.14)	£11,362.56 (2272.51)	10.2 %	£27,117.35	0.7 (0.14)	0.71 (0.02)	0.50
Humerus	0.0385 % (13, 33791)	0.0538 % (4, 8349)	1.54 (0.46)	1.33 (0.15)	£3,880.06 (776.01)	0 %	£27,117.35	0.934 (0.19)	0.71 (0.02)	0.66
Wrist	0.7824 % (264.5, 33540)	0.9757 % (82, 8271)	0.54 (0.62)	1.19 (0.15)	£2,981.60 (596.32)	0 %	£27,117.35	0.934 (0.19)	0.71 (0.02)	0.66
Other	1.7541 % (593, 33211)	2.5604 % (213, 8139)	1.03 (0.28)	1.27 (0.08)	£4,031.77 (806.35)	0 %	£27,117.35	0.934 (0.02)	0.71 (0.02)	0.66

*SE* standard error

### Model structure

The model applies a 1-year time horizon. This is justified by the fact that most costs and quality of life implications occur within the 1st year of having a fracture. Additionally, the majority of patients normally receive opioid treatment for less than 1 year [13]. However, applying this time horizon means that benefits from lower fall rates are probably underestimated.

Health states are based on the type of fracture sustained, following an approach used in previous NICE health technology assessments (see, for example, the assessment of cost effectiveness of denosumab in the prevention of osteoporotic fractures in postmenopausal women [4]). The model was validated by the Evidence Review Group involved in the submission on behalf of NICE [14]. Our model considers four types of fracture, each with its own health state: hip fracture, wrist fracture, humerus fracture and other fracture. The difference in health states in our model and the NICE model reflects the fracture risk data available in the Li et al. [10] study for the treatments under consideration.

Patients start the model in the no fracture health state; these patients are at risk of a fracture. Once a patient has a fracture, they move to the associated health state for the fracture; when in this health state, patients incur the cost and quality of life implications associated with the fracture. A patient can only incur one fracture within the 1-year time horizon.

The model does not include any opioid treatment switching following a fracture as this would have required further complexity. However, it is expected that, given the low incidence of fractures overall, switching opioids would have a minimal impact on results. Given the higher risk of fracture for patients on tramadol compared with other medications, allowing treatment switching following a fracture would slightly increase the costs for patients initially receiving tramadol.

### Efficacy inputs

To distinguish the treatments included within the model, odds ratios of fracture risk are used to estimate the difference between treatments. Li et al. [10] reports odds ratios for patients currently receiving opioid treatments including buprenorphine and tramadol in a UK population. It is assumed that the risk factors associated with buprenorphine are equivalent to transdermal buprenorphine.

Li et al. [10] is used in the base case as this study is in a UK setting. It considers evidence from the UK General Practice Research Database, which has data from 500 general physician practices; cases before the start of 1990 were excluded, resulting in a study sample of 1.7 million non-cancer patients. Li et al. [10] calculated odds ratios for hip, wrist, humerus and overall fractures. Therefore, the model uses fracture specific odds ratios for hip, wrist and humerus and applies the opioid specific overall odds ratio for the other fracture types. Two other studies report odds ratios for opioid-related risk of fractures and falls that evaluate both tramadol and buprenorphine. Vestergaard et al. [9], based in a Danish setting, looks specifically at fractures and is tested in the scenario analysis. Söderberg et al. [11], based in Sweden, is not used in the sensitivity analysis as it presents odds ratios for falls rather than fractures.

Li et al. [10] did not show statistically significant results for the risk of fractures between tramadol and buprenorphine. The sample size of the two opioid treatments is not reported; however, it is probable that, in the General Practice Research Database population, buprenorphine use is approximately a fifth of the use of tramadol. Therefore, a likely hypothesis is that the small sample size for buprenorphine is insufficient for a statistically significant result. The associated uncertainty around the point estimate in the sudy of Li et al. [10] is explored using probabilistic sensitivity analyses.

It should be noted that this paper does not directly report the figures used in the model, but presents them in a chart; therefore, the computer software GetData Graph Digitizer^®^ has been used to translate the presented chart data into numerical values. GetData Graph Digitizer cannot be completely accurate and may affect the uncertainty around the point estimates and confidence intervals.

### Number needed to harm

The model calculates the number of fractures experienced for each treatment arm based on 100,000 patients starting each treatment. The number of fractures experienced is calculated using the number needed to harm (NNH) formula [15]. The calculation is explained further in the calculation section. This results in an estimate of the numbers of patients who experience a fracture in the general population compared with the number of patients who experience a fracture using tramadol and the number of patients who experience a fracture using transdermal buprenorphine.

### Time on treatment

To estimate treatment persistence, the number of days patients are treated in a given year was estimated from a persistence study by Gallagher et al. [13], using GetData Graph Digitizer^®^. At 12 months of treatment, there are statistically significant differences in the persistence rates of patients on tramadol (17.6 %) compared with those on low-dose buprenorphine patches (18.5 %). The cost and utility implications of tramadol patients discontinuing earlier than transdermal buprenorphine patients have not been modelled. Therefore, a simplifying assumption has been made to equalize the length of treatment for both drugs at the average persistence of patients on tramadol. Over the initial 12-month treatment period, the average persistence rate for patients on tramadol, calculated from the digitized data, was 29.4 %. For the base case, this average persistence rate of tramadol in the first 12 months is used, equating to 107.2 days (29.4 % × 365 days) of treatment in a given year for both transdermal buprenorphine and tramadol. Gallagher et al. report that, post 12 months of treatment, there are no statistically significant differences in the persistence rates. In the scenario analysis, the average persistence rates for transdermal buprenorphine and tramadol post 12 months are used. This rate of 17.3 % equates to 63.2 days in a year.

### Cost inputs

The costs considered within the model are the costs of the fractures and the opioid treatments. The opioid costs were taken from the British National Formulary (BNF) and are shown in Table 2 [16]. A conversion ratio was used to equate the dose of transdermal buprenorphine to tramadol based on pain management information from the BNF [17]. The latter states that 100 mg tramadol is approximately equivalent to 10 mg morphine taken orally and that transdermal buprenorphine (BuTrans^®^ 10 µg/h 7-day patch) is the equivalent of 24 mg morphine salt daily. On this basis, one 7-day patch of transdermal buprenorphine equates to an average daily dose of 240 mg tramadol taken for 7 days. In the BNF, the closest appropriate tramadol tablet strengths are 100 and 150 mg. The model assumes an average dose of 240 mg per tramadol patient.

**Table 2:** Treatment costs, dosages and model parameters

Treatment	Pack size [16]	Pack cost [16]	Unit cost per patch/tablet	Strength	Daily dose	Daily cost	Days dosed in a year [13]	Total cost per patient
Parameter for treatment *i*	PS_i_	PC_i_	UC_i_	*S*_i_	DD_i_	DC_i_	DDY_i_	AC_i_
Transdermal buprenorphine (BuTrans)	4	£31.55	£7.88	1.68 mg (10 µg/h × 7 days)	0.24 mg (1/7 of 1 patch)	£1.13	107.18	£120.77
Tramadol (MR non-propriety)	60	£14.72	£0.25	100 mg	240 mg	£0.59	107.18	£63.11

*MR* modified release

The costs of fractures were taken from the denosumab NICE submission [4] and uplifted using the Personal Social Services Research Unit (PSSRU) [18] inflation factors to 2013 prices (inflation rate of 1.076). Costs for a wide range of fractures are reported, but a cost for wrist fracture is not. Therefore, the cost of a forearm fracture was applied as a proxy. An alternative reference for costs has been tested within the sensitivity analysis. These costs were also taken from the NICE submission [4], in which they are referred to as the costs from Stevenson et al. (2006). A proportion of patients with hip fractures enter a nursing home post fracture. The proportion of patients and the cost of the nursing home match the denosumab NICE submission. Of hip fracture patients, 10.2 % move to a nursing home, and inflated from 2010 values, each of those patients incurs £27,117.35 in nursing home costs [4, 18]. Patients were assumed to receive a full year of costs in line with the assumption around the fracture costs.

Costs associated with adverse events apart from fractures are not included in the base case but are included in the scenario analysis. Karlsson et al. [19] conducted a non-inferiority study between transdermal buprenorphine and tramadol. The results indicate a similar incidence of non-fracture adverse events for both treatments. While the overall rate of pruritus between the two treatment arms is equal, Karlsson et al. [19] find a 5.8 % incidence of application-site pruritus for transdermal buprenorphine compared with 0 % for tramadol. This is to be expected as tramadol is administered orally. As it is unclear how these patients would be treated, and due to the lack of available evidence, it is assumed in scenario analysis that, conservatively, the proportion of patients experiencing application site pruritus incur the cost of a doctor’s appointment of £53 [18].

Karlsson et al. [19] find transdermal buprenorphine to be non-inferior to tramadol in terms of pain control. Therefore, analgesic pain control is not explicitly modelled. They also find that the average dose of transdermal buprenorphine to manage pain control stabilized between 10 and 12 µg/h. Therefore, the model uses one 10 µg/h 7-day patch for the transdermal buprenorphine dosage. The formulation of tramadol was derived from Karlsson et al. [19]; patients received twice-daily modified release tramadol tablets.

### Utility input data

Utility input data are derived from the NICE submission [4], where a fracture-specific utility multiplier was applied to background utility. To be in line with the NICE submission, background utility is taken from the general population reported by Kind et al. [20]. A utility value of 0.71 is reported for women aged 75+. The utility multipliers used within the model are reported in Table 1 [4]. The utility multipliers are applied for 1 year, which is in line with the NICE submission [4].

### Model calculations

In depth calculations are provided for the different parts of the model, with the aim of making the model as transparent as possible and, subsequently, making the model reproducible.

For these calculations, the following definitions are used throughout:

*i* = type of fracture, where 1 refers to hip, 2 refers to humerus, 3 refers to wrist, and 4 refers to other.

*j* = treatment, where 1 refers to transdermal buprenorphine, and 2 refers to tramadol.

#### Number needed to harm calculations

NNH_*ij*_ = Number needed to harm, where *i* refers to fracture type and *j* refers to the treatment

OR_*ij*_ = Odds ratio of fracture *i* based on treatment with *j*

*F*_*i*_ = General population risk from Singer et al. [12] for fracture *i*

*P* = Size of the cohort (100,000 patients)

AF_*ij*_ = Additional number of fractures resulting from treatment *j* compared with general population per population

$$ {\text{NNH}}_{ij} = \left( {\left( {F_{i} \; \times \;\left( {{\text{OR}}_{ij} - 1} \right) + 1} \right)/\left( {F_{i} \; \times \,\left( {{\text{OR}}_{ij} - 1} \right)\; \times \;\left( {1 - F_{i} } \right)} \right)} \right) $$

$$ {\text{AF}}_{ij} = P/{\text{NNH}}_{ij} $$

#### Fracture calculations

TF_*j*_ = Total number of fractures for treatment *j* and fracture type *i*

$$ {\text{TF}}_{j} = \mathop \sum \limits_{i = 1}^{i = 4} ({\text{AF}}_{ij} + F_{i} \; \times \;P) $$

#### Cost calculations

AC_*j*_ = Cost of treatment course

PC_*j*_ = Cost per pack of treatment *j*

PS_*j*_ = Tablets/patches per pack of treatment *j*

S_*j*_ = Strength of the pack (mg)

SC_*j*_ = Total societal costs

DD_*j*_ = Daily dose for treatment *j*

*P* = Size of the cohort (100,000 patients)

*C*_i_ = Cost of fracture *i*

PNH_*i*_ = Proportion of patients moving to a nursing home post fracture

CNH = Annual cost of a nursing home

TCF_*j*_ = Total cost of fractures for treatment *j*

DDY_*i*_ = Number of days of treatment

TC_*j*_ = Total cost for treatment *j*

$$ {\text{AC}}_{j } = \frac{{\frac{{{\text{PC}}_{j} }}{{{\text{PS}}_{\text{j}} }}}}{{{\text{S}}_{j } }}\; \times \;{\text{DD}}_{j } \; \times \;{\text{DDY}}_{i } $$

$$ {\text{SC}}_{j } = \mathop \sum \limits_{i = 1}^{i = 4} ({\text{AF}}_{ij} + F_{i} \; \times \;P)\; \times \;{\text{PNH}}_{i } \; \times \;{\text{CNH}} $$

$$ {\text{TCF}}_{j} = \mathop \sum \limits_{i = 1}^{i = 4} ({\text{AF}}_{ij} + F_{i} \; \times \;P)\; \times \;C_{i} $$

$$ {\text{TC}}_{j} = {\text{AC}}_{j} \; \times \;P + {\text{TCF}}_{j} + {\text{SC}}_{j } $$

#### QALY calculations

*U*_*i*_ = Utility multiplier for fracture *i*

BU = Baseline utility

TQ_*j*_ = Total utility associated with treatment *j*

$$ {\text{TQ}}_{j} = P\; \times \;{\text{BU}} - \mathop \sum \limits_{i = 1}^{i = 4} (({\text{AF}}_{ij} + F_{i} \; \times \;P) - ({\text{AF}}_{ij} + F_{i} \; \times \;P) \; \times \;U_{i} )\; \times \;{\text{BU}} $$

#### Result calculations

TQ_*j*_ = Total utility associated with treatment *j*

TC_*j*_ = Total cost for treatment *j*

ICER = Incremental cost-effectiveness ratio

$$ {\text{ICER}} = \frac{{{\text{TC}}_{1 } - {\text{TC}}_{2 } }}{{{\text{TQ}}_{1 } - {\text{TQ}}_{2 } }} $$

### Model outputs

Model results are presented in terms of the ICER as well as the calculated number of incremental fractures compared with the general population. In line with NICE guidelines the willingness to pay threshold per QALY is £20,000, this threshold is a conservative assumption as NICE have stated that treatments with an ICER of £20,000–30,000 may be considered cost-effective depending on additional criteria [21].

### Sensitivity analysis

#### Deterministic sensitivity analyses

Deterministic one-way sensitivity analyses were used for the parameters that have uncertainty around them within the model. Table 1 shows the different category of parameters that are included in the analysis with the associated uncertainty and distribution. Where possible the uncertainty was taken from published evidence; where this is not possible a plausible range was used (20 % variation of standard error).

The one-way sensitivity was plotted in net marginal benefit per patient, using a threshold of £20,000; hence, a net marginal benefit greater than 0 was considered cost-effective at a threshold of £20,000 [21].

#### Scenario analyses

Scenario analysis was used to test uncertainty, alternative references or to validate assumptions within the model. Five alternative scenarios were included in the model:

- An alternative reference for the fracture odds ratios. The study by Li et al. [10] was used in the base case. The scenario analysis used a study by Vestergaard et al. [9].
- A proportion of patients receiving buprenorphine experience application-site pruritus; 5.8 % of patients receiving transdermal buprenorphine experience application-site pruritus [19].
- An alternative reference for the cost of fractures within the model was tested. The base case used the same cost reference as reported in the base case of the denosumab NICE submission, and as with that submission, alternative costs as found in the Stevenson et al. paper were tested [4].
- An alternative population is tested within the model; data are available from the Singer et al. [12] paper for a population of women aged 85+; as this population is of even higher risk, the effects in this population were modelled.
- A scenario with fewer treatment days in the year was tested to represent the findings from the study by Gallagher et al. [13] at 12 months.

#### Probabilistic sensitivity analysis

A probabilistic sensitivity analysis (PSA) is included to investigate uncertainty within the model further. For each uncertain parameter in the model, a value was sampled from a distribution around the mean based on the uncertainty shown in Table 1. This was repeated for 10,000 iterations.

## Results

### Base case results

For a population of 100,000 women aged 75 and older, patients treated with tramadol would suffer in excess of 1,000 extra fractures compared with both a general population and a population treated with transdermal buprenorphine. The full results of the number of fractures are presented in Table 3. The incremental fractures resulted in transdermal buprenorphine being a cost-effective alternative to tramadol at a threshold of £20,000. The total costs of fractures per 100,000 women are shown in Table 4, and the base case results are presented in Table 5.

**Table 3:** Expected number of fractures per 100,000 women

Fracture	General population		Tramadol		Transdermal buprenorphine	
	Total fractures	Total QALY	Total fractures	Total QALY	Total fractures	Total QALY
Hip	1,529	760	1,979	984	1,830	909
Humerus	39	26	51	34	59	39
Wrist	782	519	930	617	424	281
Other	1,754	1,163	2,217	1,470	1,806	1,197
Total fractures	4,104	2,468	5,177	3,104	4,119	2,427
Remaining patients	0	68,086	0	67,324	0	68,076
Total	4,104	70,554	5,177	70,429	4,119	70,503
Incremental compared to general population	–	–	1,073	−125	14	−51
Incremental compared to tramadol	–	–	–	–	−1058	74

*QALY * quality-adjusted Life Year

**Table 4:** Total costs of fractures per 100,000 women

Fracture	General population			Tramadol			Transdermal buprenorphine		
	Total cost of fractures	Total treatment cost	Total cost	Total cost of fractures	Total treatment cost	Total cost	Total cost of fractures	Total treatment cost	Total cost
Hip	£21,608,173	£0	£21,608,173	£27,962,329	£124,899	£28,087,227	£25,850,736	£239,018	£26,089,754
Humerus	£149,382	£0	£149,382	£198,653	£3,231	£201,884	£230,001	£6,183	£236,184
Wrist	£2,332,801	£0	£2,332,801	£2,771,912	£58,669	£2,830,582	£1,264,263	£112,276	£1,376,538
Other	£7,072,131	£0	£7,072,131	£8,939,270	£139,923	£9,079,192	£7,280,464	£267,770	£7,548,234
Total fracture	£31,162,487	£0	£31,162,487	£39,872,164	£326,722	£40,198,885	£34,625,463	£497,418	£35,122,881
Remaining patients	£0	£0	£0	£0	£5,984,037	£5,984,037	£0	£11,579,472	£11,579,472
Total	£31,162,487	£0	£31,162,487	£39,872,164	£6,310,758	£46,182,922	£34,625,463	£12,076,889	£46,702,352
Incremental cost compared to general population	–	–	–	£8,709,676	£6,310,758	£15,020,434	£3,462,976	£12,076,889	£15,539,865
Incremental cost compared to tramadol	–	–	–	–	–	–	−£5,246,701	£5,766,131	£519,430

**Table 5:** Base case results per 100,000 women

Treatment	Total cost	Total QALY	Incremental cost	Incremental QALY	ICER
Transdermal buprenorphine	£46,702,352	70,503			
Tramadol	£46,182,922	70,429	£519,430	74	£6,979

*ICER * incremental Cost-effectiveness Ratio; *QALY* quality-adjusted life year

These results suggest that although tramadol lowers treatment costs, the reduction in fractures means that transdermal buprenorphine has an incremental cost of £5.19 per patient, and £519,430 for the full cohort of 100,000 patients. When this incremental cost per incremental QALY is considered, the ICER is well below the generally accepted £20,000 per QALY threshold in the UK.

### Deterministic sensitivity analyses

**Fig. 2:**
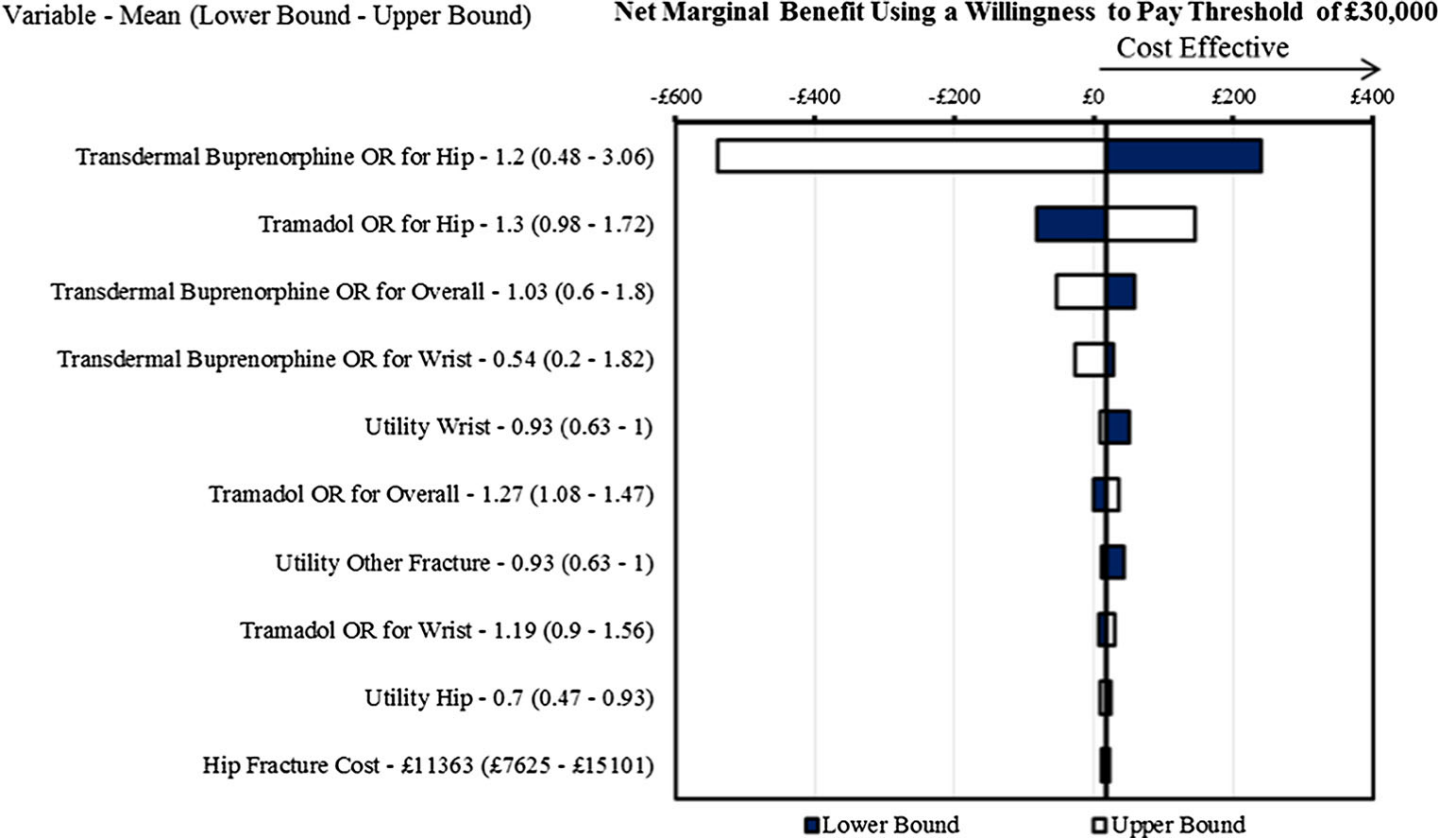
One-way sensitivity analysis comparing transdermal buprenorphine to tramadol—per patient

The one-way sensitivity analysis found the model to be most sensitive to the odds ratios for both the transdermal buprenorphine and tramadol (Fig. 2). Baseline utility is also an important factor within the model. Only the odds ratios of transdermal buprenorphine and tramadol resulted in transdermal buprenorphine not being cost-effective.

### Scenario analyses

The scenario analysis results are presented in Table 6. The analysis shows that the model is robust with all ICERs below a £30,000 threshold. When using the odds ratios from Vestergaard et al. [9], transdermal buprenorphine is cost saving.

**Table 6:** Scenario analysis per 100,000 women

Scenario	Incremental cost	Incremental QALY	ICER
Odds ratio reference [9]	−£28,534,354	491	Tramadol is dominated
Adverse events (application-site pruritus) [18]	£613,103	74	£8,237
Stevenson costs [4]	£2,005,040	74	£26,939
85+ patient population [12]	−£2,635,436	120	Tramadol is dominated
Days treated in a given year (63.2 days for both treatments) [13]	−£1,895,050	74	Tramadol is dominated

*ICER* incremental cost-effectiveness ratio, *QALY* quality-adjusted life year

### Probabilistic sensitivity analysis

**Fig. 3:**
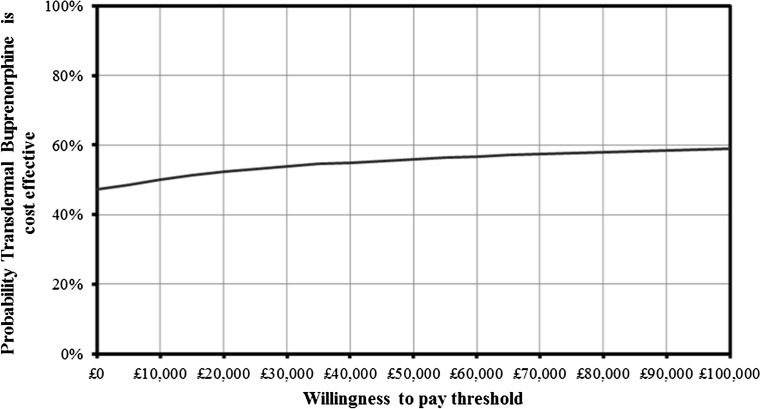
Cost-effectiveness acceptability curve

At a willingness-to-pay threshold of £30,000, it was estimated that transdermal buprenorphine had a 54 % probability of being cost-effective compared to tramadol and a 52 % probability of being cost-effective at a threshold of £20,000. It was also estimated that there was a 47 % probability that transdermal buprenorphine was cost saving compared with tramadol (Fig. 3).

## Discussion

This study investigated the health system burden of fractures related to the use of tramadol in a moderate pain setting in the UK. In total, tramadol was associated with 1,073 additional fractures compared with the general population, whereas transdermal buprenorphine was associated with a similar number of fractures to the general population. The high level of additional fractures due to use of tramadol led to an incremental cost of fractures of £8,709,676 compared to a general population of 100,000 women.

A cost–utility model based on a publicly available NICE submission of denosumab [4], updated with a recent study of risk of fractures [10], was developed. Despite the lower medication cost of tramadol relative to transdermal buprenorphine, the costs and utility effects of extra fractures expected to result from its use meant that the cost–utility model estimated an ICER of £6,979 for transdermal buprenorphine compared with tramadol. It is therefore likely transdermal buprenorphine would be considered cost-effective at the UK willingness-to-pay threshold of £20,000 per QALY.

The robustness of the model result is supported by the PSA, with a 52 % probability of transdermal buprenorphine being cost-effective at a threshold of £20,000 compared with tramadol. This probability was not higher, despite the low point estimate of cost effectiveness, because of the wide confidence intervals around the buprenorphine odds ratios in the UK-based study by Li et al. [10], which was used as the source of odds ratios in the base case. Tramadol odds ratios were narrow in comparison and showed a statistically significant higher overall fracture risk compared with the general population. The deterministic one-way sensitivity analysis demonstrated that the ICER was relatively insensitive to individual changes in the model parameters, with the exception of the buprenorphine odds ratios, as reported by Li et al. [10], which were associated with high uncertainty. However, other scenario analyses tested odds ratios and their associated uncertainties from alternative sources relating to other countries. In the scenario analysis, transdermal buprenorphine was found to be cost-effective in all cases and, in some scenarios, dominated tramadol with cost savings to the health system.

The model and associated inputs have some limitations. Firstly, the odds ratios applied were for the general opioid population and were not specific to the high-risk group considered in the model; therefore, the assumption had to be made that the ratios would be consistent over the range of absolute risk involved. However, some similarities can be seen between the population considered in Li et al. and the high-risk group in the model (the Li et al. [10] population has a mean age of 62.4 and 76.6 % female). Li et al. also comment that there is no statistically significant difference in age stratifications.

Secondly, the study of Li et al. [10] did not show a statistically significant result for the risk of fractures for buprenorphine relative to other opioids. However, the studies by Vestergaard et al. and Söderberg et al. [9, 11] provided statistically significant results in which tramadol patients had an increased risk of fractures and falls relative to buprenorphine. As shown in the scenario analysis, the use of Vestergaard et al. [9] results in tramadol being dominated by buprenorphine. The lack of statistical significance for the buprenorphine result in Li et al. is potentially due to a smaller sample size of patients receiving buprenorphine.

The data taken from Singer et al. [12] also represents a limitation of the model because the general population reported is likely to include some opioid use. A non-opioid population would be expected to have a lower risk of fracture; however, because opioid use would only effect a small percentage of patients, it is assumed that the data from Singer et al. is reasonably reflective of a non-opioid population.

A further limitation of the model is the simplifying assumption of a 1-year time horizon. The model assumes that all fractures happen within the 1st year of treatment and all cost and quality of life decrements that follow a fracture also occur within this time frame. This approach may overestimate the total fracture cost that occurs in a year; in reality the cost may spread over a longer time frame. However, patient utility impact may be underestimated as some of the more serious fractures may have quality of life implications for several years.The duration of the model also meant that the potential impact of severe fractures influencing overall survival was not modelled. Hip fracture in the elderly, in particular, is associated with increased mortality risk [22].

## Conclusion

In a patient group treated with tramadol, there is an increased risk of fracture-related healthcare costs compared to both a general population and a group treated with transdermal buprenorphine. Cost savings and quality of life gains due to lower fracture rates result in treatment with transdermal buprenorphine being cost-effective compared to tramadol at an ICER threshold of £20,000.
